# Hydrogen sulfide inhibits ethylene-induced petiole abscission in tomato (*Solanum lycopersicum* L.)

**DOI:** 10.1038/s41438-019-0237-0

**Published:** 2020-02-01

**Authors:** Danmei Liu, Jianing Li, Zhuowen Li, Yanxi Pei

**Affiliations:** 1 College of Life Science, Shanxi University, Taiyuan, 030006 China; 2 Shanxi Key Laboratory for Research and Development of Regional Plants, Taiyuan, 030006 China

**Keywords:** Plant physiology, Plant molecular biology

## Abstract

Abscission is a dynamic physiological process that is ubiquitous in plants and can also be an essential agronomic trait in crops, thus attracting attention from plant growers and breeders. In general, the process of plant organ abscission can be divided into four steps, among which the step to obtain the competence to respond to abscission signals (step 2) is the most complex; however, the molecular mechanism underlying this process remains unclear. In this study, we found that hydrogen sulfide (H_2_S) inhibited the abscission of the tomato petiole in a dose-dependent manner, and the abscission of the petiole was accelerated when an H_2_S scavenger was applied. Further enzymatic activity and gene expression analyses showed that H_2_S suppressed the activity of enzymes capable of modifying the cell wall by inhibiting the usual upregulation of the transcription of the corresponding genes during the abscission process but not by affecting the activities of these enzymes by direct posttranslational modification. H_2_S treatment upregulated the expression levels of *SlIAA3* and *SlIAA4* but downregulated the transcription of *ILR-L3* and *ILR-L4* in the earlier stages of the abscission process, indicating that H_2_S probably functioned in the second step of the abscission process by preventing the abscission zone cells from obtaining the competence to respond to abscission signals by modulating the content of the bioactive-free auxin in these cells. Moreover, similar H_2_S inhibitory effects were also demonstrated in the process of floral organ abscission and anther dehiscence in other plant species, suggesting a ubiquitous role for H_2_S in cell separation processes.

## Introduction

Abscission is a critical process at different stages during the life span of a plant and ensures unwanted organs are shed from the main body of the plant^1^. It is also an important agronomic trait with major implications in terms of yield, quality, and postharvest storage in some crops^2,3^. Abscission always occurs in a morphologically distinct area called the abscission zone (AZ). AZs are usually located at predetermined positions at the base of most plant organs, encompassing several layers of small, densely arranged cytoplasmic cells^4^. The formation of an AZ is the first step of the abscission process. Thereafter, through the perception of environmental and developmental signals, AZ cells attain the competence to respond to abscission signals (step 2) before the activation and execution of abscission (step 3)^1^. Finally, a protective layer is formed distal to the separation layers to protect the organs remaining on the plant from pathogen infection and water loss (step 4)^4–6^.

As an essential physiological process that has been studied for many decades, many classical phytohormones have been shown to exert an influence over abscission, with ethylene and auxin reported to play central roles in the abscission process. Ethylene is regarded as an accelerator, whereas auxins decelerate abscission^2,5,7^. Evidence has shown that organ abscission is delayed in ethylene-insensitive mutants of many plant species^1,8,9^, while auxins have been shown to inhibit abscission by rendering AZ cells insensitive to ethylene^10,11^. Modern agriculture manipulates the process of abscission to the growers’ advantage by applying vinylogous, ethylene inhibitors or synthetic auxins to thin excess flower numbers early in the season and hence avoid overcropping or to prevent preharvest fruit drop later in the season in orchards^1^.

Abscission is a process in which the cell adhesion within AZs is deliberately dissolved. The cell wall surface shared by adjacent cells is the main medium that maintains the adhesion between cells^12,13^, and nearly 90% of the cell wall mass consists of polysaccharides (mainly pectin, cellulose, hemicellulose, etc.). During the process of abscission, it has been reported that the expression and activity of cell-wall-modifying enzymes, which hydrolyze the cell wall and the middle lamella, increase markedly^12,14^. Among these enzymes, cellulases (Cels, endo-1,4-beta-glucanases), polygalacturonases (PGs), and expansins are essential members, and their functions have been investigated in cell separation processes^14–16^. It has been shown that silencing tomato PGs (TAPGs) greatly delayed abscission, even in the presence of ethylene, a major abscission accelerator^14^. Furthermore, the antisense suppression of tomato endo-1,4-beta-glucanase mRNA also increased the break strength of AZs^17^, indicating the principal roles of Cels and PGs in the process of cell wall degradation during abscission.

Hydrogen sulfide (H_2_S) is a newly discovered gasotransmitter^18^. Recently, increasing evidence has demonstrated that H_2_S possesses multiple physiological functions during the development of both animals and plants^19,20^. In tomato, limited studies have shown that H_2_S is involved in the auxin- or methane-induced lateral root formation^21,22^, the modulation of fruit ripening via increasing antioxidant activity^23^, and the alleviation of stress caused by salt or excess nitrate through altering the redox status of the stressed plants^24,25^.

In this study, we explored the function of H_2_S during the processes of plant organ abscission, especially with respect to petiole abscission in tomato, and investigated the mechanism underlying the inhibitory effects of H_2_S on abscission. Our findings extended the understanding of the physiological roles of H_2_S and provided further insights into the regulatory networks underlying the abscission process in plants.

## Materials and methods

### Plant materials and growth conditions

Seeds of the tomato cv. MicroTom were kindly provided by Dr. Yongfu Fu from the Institute of Crop Sciences, Chinese Academy of Agricultural Sciences, Beijing, China. All tomato plants were cultivated in a growth chamber under 24 °C/16-h day and 18 °C/8-h night conditions.

### Petiole abscission assay

Explants, including a 1.0-cm length stem connected to an adjacent 1.0-cm length petiole, were prepared from the nodal region of tomato plants with five to seven leaves prior to the reproductive stage. The explants were then inserted into 0.8% agar in a 400-ml growth jar (five explants per jar, five replicates) and placed in the same growth chamber with the tomato plants. The explants were then divided into three groups: the ethylene treatment group, which was fumigated with ethylene (supplied by 40 μl of 1 M ethephon solution), the H_2_S–ethylene treatment group, which was treated with both ethylene and H_2_S (supplied by 16 μl of 1 M sodium hydrosulfide (NaHS)), and the hypotaurine (HT)–ethylene treatment group, which was treated with both ethylene and the H_2_S scavenger HT (1 mM). For both ethylene and H_2_S treatment, their donors were not applied to the explants directly. The donor solutions were placed in sterile caps of centrifuge tubes, which were placed in the same growth jar with the explants, and the ethylene or H_2_S generated by the donor solutions was used to fumigate the explants. To accelerate the release rate of ethylene from ethephon, ethephon was diluted with a sodium hydroxide (NaOH) solution. The HT solution was applied directly, and explants in the other treatment groups were treated with double distilled water as a control. The petiole abscission of these three groups was recorded every 4 h and photographed at the time points 0, 12, 24, 36, and 48 h after initiation of the treatment. The abscission rates of the tomato petioles were also analyzed at different concentrations of H_2_S (provided by 0, 4, 8, 12, 16, 20, or 40 μl of 1 M NaHS solution) after treatment for 48 h. An abscission event was defined as spontaneous petiole detachment or detachment in response to a gentle vibration applied to the distal part of the petiole^26^.

### Triple response assay

The tomato seeds were surface-sterilized with 95% ethanol for 30 s and 20% bleach for 20 min, rinsed with distilled water 3–5 times, and placed in 12-cm petri dishes with two layers of wet filter papers to germinate. When the roots of the seedlings began to emerge, the seedlings were divided into four treatment groups (ethylene, H_2_S, ethylene plus H_2_S, and the nontreatment control group). Ethylene and H_2_S were applied as described above in the form of fumigation with ethephon (40 μl of 1 M solution) and NaHS (16 μl of 1 M solution) as their donors, respectively. The phenotype of these seedlings was observed and photographed after growth in the dark for 4 days, and the hypocotyl lengths of these seedlings under different treatments were measured.

### H_2_S content assay

For the H_2_S content assay, the AZ tissues prepared as mentioned above from the explants treated with different concentrations of H_2_S for 24 h were used to analyze the H_2_S content as described previously^27^. The petiole AZ tissues were first ground into homogenate with 1 ml of phosphate buffer solution (pH 7.0, 50 mM) containing 0.1 M EDTA and 0.2 M ascorbic acid. Then the homogenate was mixed with 1 ml of 1 M HCl and placed in a closed vial to release H_2_S. The released H_2_S was absorbed by a 1% (w/v) Zn(AC)_2_ (0.5 ml) trap, which was also located in the vial. After incubation for 30 min, 100 μl of 20 mM *N*,*N*-dimethyl-p-phenylenediamine and 100 μl of 30 mM FeCl_3_ were added to the Zn(AC)_2_ solution. After incubation in the dark for 15 min, the absorbance was measured at 670 nm to determine the level of H_2_S produced.

### Total RNA extraction and quantitative PCR (qPCR)

Total RNA was extracted using RNAiso Plus (TaKaRa, Shiga, Japan) according to the manufacturer’s instructions and then reverse transcribed into cDNA using an All-In-One RT MasterMix (abm, Nanjing, China), in which a finely balanced ratio of oligo(dT)s and random primers was provided. A 7500 Fast Real-Time PCR system (Applied Biosystems, Foster City, USA) was used to perform the real-time qPCR, and a *SAND* family gene (Solyc03g115810) was used as an endogenous control^28^. The relative expression levels of genes were determined using the 2^−ΔΔCt^ method^29^. Each analysis was conducted with three biological and three technical replicates, and the primer sequences are listed in Table S1.

### PG activity assay

The petiole explants were prepared and treated with ethylene or ethylene plus H_2_S as described above. The treated AZ tissues were then cut off and placed in a precooled mortar with 1 ml of extraction buffer (50 mM acetate buffer, pH 4.4) and ground into a homogenate. After incubation for 3 h at 4 °C, the homogenate was centrifuged (3000 rpm for 20 min), and the supernatant (“enzyme extract”) was collected for the following analysis. Before the initiation of the reaction, an aliquot (250 μl) of enzyme extract was preheated at 37 °C for 3 min, and then 500 μl 1% polygalacturonic acid solution (in acetate buffer, pH 4.0) was added and mixed with the extract. The sample was then incubated at 37 °C for 30 min, after which 500 μl DNS (3,5-dinitro salicylic acid; Solarbio, Beijing, China) was added to detect the galacturonic acid generated. The reaction mixture was then boiled for 5 min, quickly cooled, and 1.25 ml distilled water was added to the reaction system before measuring the absorbance of the reaction mixture at 540 nm to determine the concentration of the galacturonic acid produced. D-(+)-galacturonic acid was used as the standard sample to construct the standard curve.

### Cel activity assay

The preparation and enzyme extraction of the petiole AZ tissues was similar to that described above for PG activity detection, except that the extraction buffer was 100 mM acetate buffer, pH 4.6, and the substrate in this assay was sodium carboxymethyl cellulose (1% w/v in 100 mM acetate buffer, pH 4.6). The incubation temperature for the reaction in this assay was 40 °C. DNS was again used in this analysis to detect the reducing sugar generated, and glucose standards were used to construct the standard curve.

### Prokaryotic expression, protein purification, and enzymatic activity assay

To express *TAPG4* and *Cel5* in *Escherichia coli*, the mature coding region sequences of these two genes were first cloned into the expression vector pCold and then transformed into *E. coli* BL21 to achieve exogenous protein expression. The expressed recombinant proteins were then purified using a Ni-NTA Sefinose Resin (Sangon Biotech, Shanghai, China), following the manufacturer’s instructions, and the purity was confirmed by 10% sodium dodecyl sulfate-polyacrylamide gel electrophoresis. The purified proteins were then used to detect the activities of PG (TAPG4) and Cel (Cel5), respectively. The protein preparation isolated from the empty pCold vector was used as the negative control. For H_2_S treatment, its donor NaHS was combined with the protein solutions directly at different concentrations (0, 10, 20, 30, 40 μM) as reported previously^30^.

### Abscission or dehiscence analysis in other plant species exposed to H_2_S

Flowering stems of rose (cv. Yingxing) and lily (cv. Suoerbang) were obtained from the local flower market in Taiyuan, China. Each flower material was divided into two groups that were exposed to either ethylene or the ethylene–H_2_S combination using the same concentrations and the treatment method as described earlier. For the floral organ abscission analysis of roses, the abscission rate was recorded every 3 h and photographed every 6 h after treatment. For the anther dehiscence assay of lilies, all treatments were performed for 22 h in airtight containers, and then the treatments were removed and the anthers were exposed to the air. At this time point, the anther in the ethylene-treated group immediately began to open after the container was removed. Then the dehiscence rates of these anthers were recorded every 20 min after being exposed to the air.

### Statistical analysis

For each sample and assay, three biological and three technical replicates were performed, and the summary statistics of the data are presented as the mean ± standard error (SE). Duncan’s multiple range test of the SPSS 19.0 software (IBM, Armonk, NY, USA) was used for all data analyses, with *P* < 0.05 as the threshold level for significance.

## Results

### H_2_S inhibited petiole abscission

**Fig. 1:**
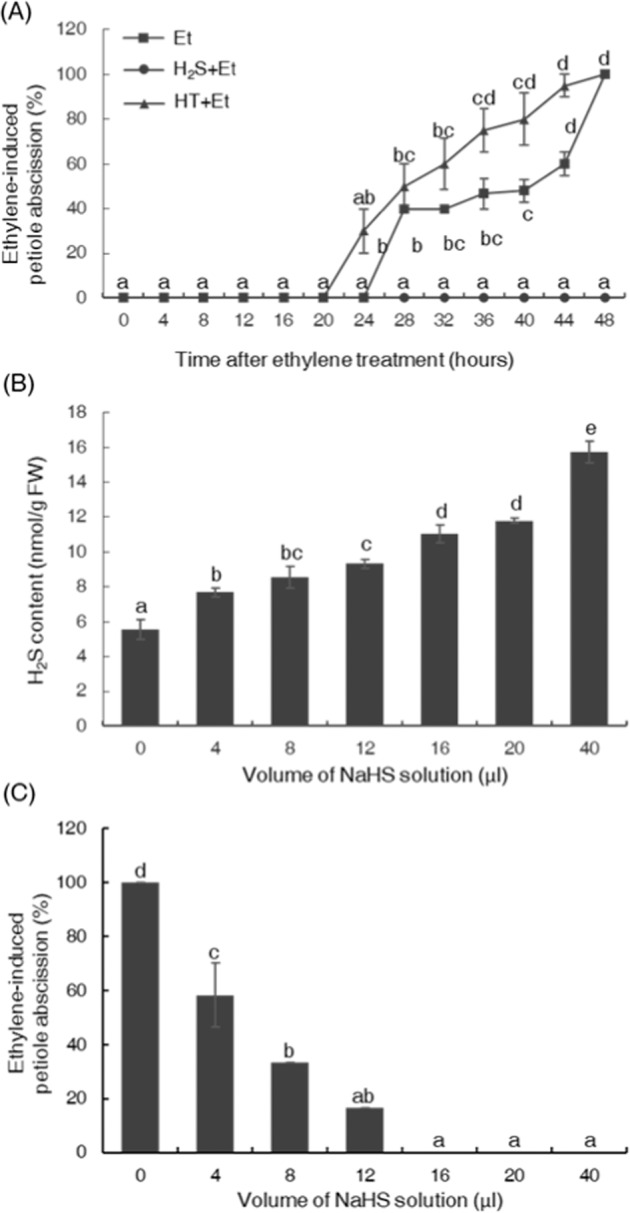
H_2_S inhibited the process of petiole abscission in tomato. **a** Hydrogen sulfide (H_2_S) and its scavenger hypotaurine (HT) acted antagonistically during petiole abscission. H_2_S was supplied by sodium hydrosulfide (16 μl of 1 M NaHS solution), with HT (1 mM) as the H_2_S scavenger. **b** The endogenous H_2_S content of the tomato petiole abscission zone tissues fumigated with different concentrations of H_2_S provided by different amounts of 1 M NaHS solutions. The H_2_S contents were measured after incubation with H_2_S gas for 24 h. **c** The inhibitory effects of H_2_S on petiole abscission are concentration dependent. The petiole abscission rates were recorded after treatment for 48 h.

To investigate the effect of H_2_S on the abscission process of tomato petioles, H_2_S, supplied by NaHS, was applied to the tomato petiole explants at its physiologically active concentration^14^, and any petiole abscission was recorded every 4 h. To synchronize the abscission process of different tomato petiole explants, exogenous ethylene (supplied by ethephon solution) was also applied, as in the natural petiole abscission system; however, the deviation was too large, and the results were hard to replicate (Fig. S1). The results showed that, in the ethylene treatment group, the tomato petioles started to abscise at 28 h after the initiation of the experiment, nearly half of the petioles had abscised by 36 h of treatment, and all the tomato petioles abscised by 48 h of treatment (Fig. 1a). However, in the presence of H_2_S, the petiole abscission was greatly inhibited; at 48 h after the experiment was set up, petioles in this group (H_2_S–ethylene group) did not show any sign of abscission (Fig. 1a). To assess the function of endogenous H_2_S, the H_2_S scavenger HT was applied to the tomato petiole together with ethylene. As a result, H_2_S scavenging accelerated abscission, as abscission in this group started at 24 h, and >60% of the petioles had abscised by 36 h treatment (Fig. 1a).

To rule out the possibility that H_2_S might affect petiole abscission by inhibiting the release of ethylene from ethephon, a triple response analysis was conducted under treatments with different combinations of ethylene and H_2_S. The results showed that the hypocotyls of the tomato seedlings treated with ethylene or H_2_S–ethylene exhibited the same degree of shortening compared with the nontreatment group, indicating that H_2_S did not inhibit the release of ethylene from ethephon (Fig. S2).

To further investigate the relationship between H_2_S and the inhibition of petiole abscission, we compared the abscission rate under different H_2_S levels provided by different amounts of NaHS solutions (Fig. 1b). Our data showed that the inhibitory effect of H_2_S on petiole abscission was concentration dependent. When the amounts of the H_2_S donor (NaHS) solutions increased from 0 to 16 μl, the abscission rate decreased from 100% to 0%, indicating that H_2_S was a novel regulator of the process of tomato petiole abscission (Fig. 1c).

### H_2_S inhibited both PG and Cel activities in the petiole AZ

**Fig. 2:**
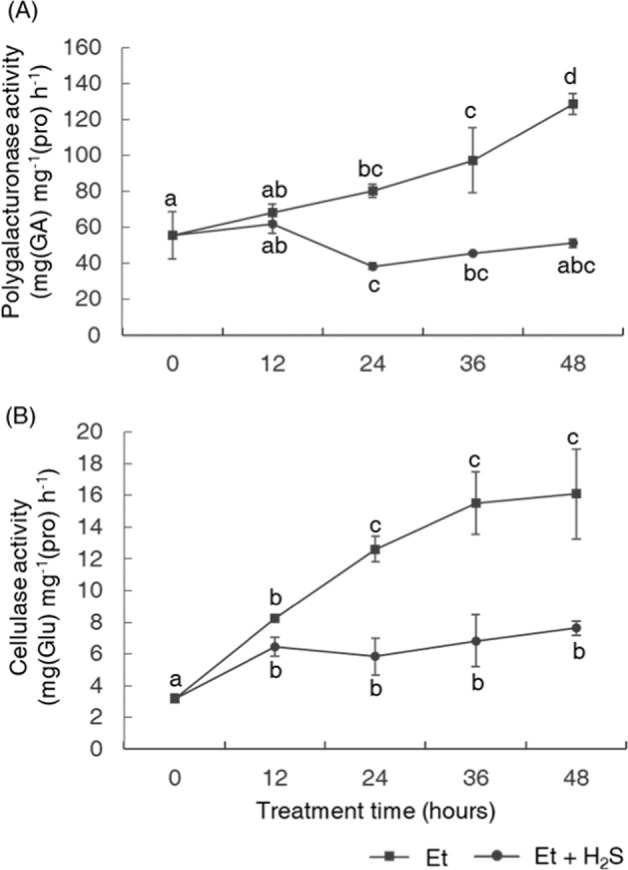
H_2_S suppressed the activities of cell-wall-modifying enzymes during petiole abscission. H_2_S suppressed the ethylene-induced increase in the enzymatic activities of polygalacturonase (**a**) and cellulase (**b**) in the AZs of tomato petioles. The activities of the enzymes were determined at 0, 12, 24, 36, and 48 h after the initiation of the assay. Data are presented as the mean ± SE (*n* = 3), and any two samples with a common letter are not significantly different (*P* > 0.05).

Cell-wall-modifying enzymes play important roles in abscission. To determine the mechanism by which H_2_S inhibited petiole abscission, we measured both PG and Cel activities in petiole AZ tissues during petiole abscission under H_2_S treatment. The results showed that, in the ethylene treatment group, the activities of both enzymes increased dramatically as abscission progressed (Fig. 2). However, in the H_2_S–ethylene treatment group, PG activity remained at a significantly lower level during treatment (Fig. 2a), while the activity of Cel increased only slightly over the same time period (Fig. 2b).

### H_2_S suppressed the upregulation of genes encoding cell-wall-modifying enzymes

**Fig. 3:**
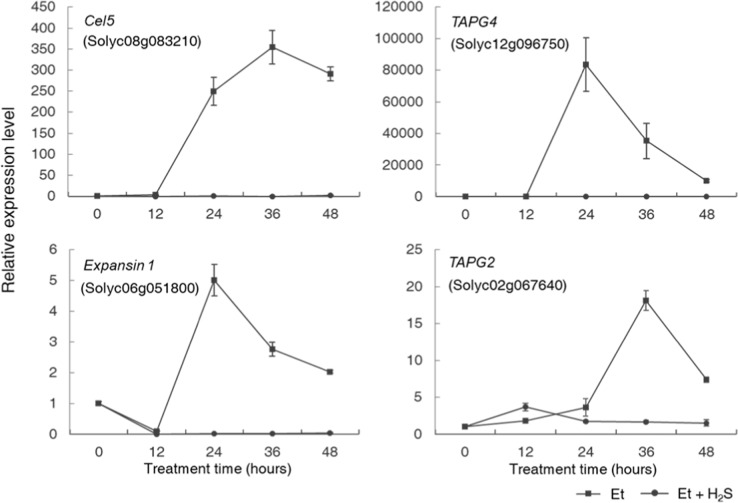
H_2_S inhibited the ethylene-induced upregulation of the expression of genes encoding cell-wall-modifying enzymes in the tomato petiole AZ. Changes in the expression levels of *Cel5*, *TAPG4*, *Expansin1*, and *TAPG2* in AZ tissues were monitored during petiole abscission under both ethylene (square) and ethylene–hydrogen sulfide (H_2_S) (circle) treatments with 12-h intervals. Data are presented as the mean ± SE (*n* = 3). The *SAND* gene was used as an internal control.

As H_2_S inhibited the activities of cell-wall-modifying enzymes in the petiole AZs, we investigated whether this was due to an effect of H_2_S on the transcription of the genes encoding these enzymes. We monitored the expression levels of the genes *Cel5*, *TAPG2*, *TAPG4*, and *Expansin1*, which have been reported to be involved in the process of abscission in tomato and encode cell-wall-modifying enzymes^16,31,32^. In the ethylene treatment group, the expression levels of all four genes increased sharply and significantly after 12 h, reaching their expression peaks at 24 or 36 h (Fig. 3). However, in the H_2_S–ethylene treatment group, the upregulation of all four genes was inhibited, indicating that H_2_S significantly suppressed the transcription of these genes associated with the biosynthesis of cell-wall-modifying enzymes in petiole abscission (Fig. 3).

### Effect of H_2_S on PG and Cel activities in vitro

**Fig. 4:**
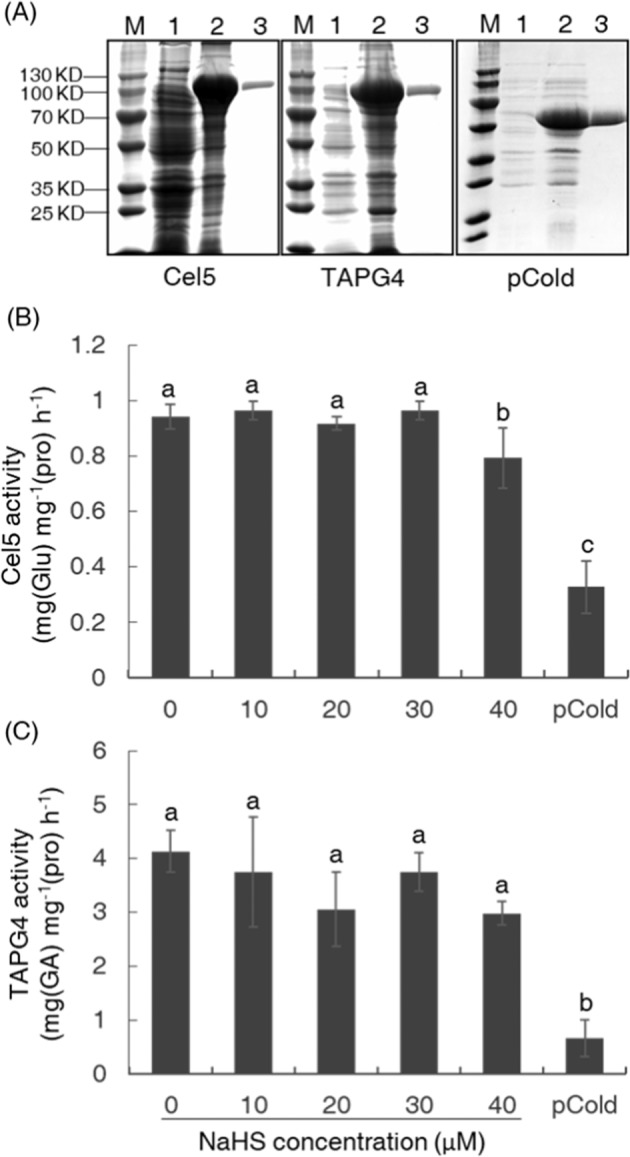
H_2_S did not inhibit the activities of cell-wall-modifying proteins directly. Detection of the purified exogenously expressed Cel5 and TAPG4 proteins (**a**) and the effects of NaHS concentrations on their activities in vitro (**b**, **c**). M, protein molecular weight ladder; 1, proteins extracted from uninduced *Escherichia coli*; 2, proteins extracted from isopropyl β-d-thiogalactoside (IPTG)-induced *E. coli*; 3, the purified protein. Protein produced by the empty pCold vector was used as a negative control. Data are presented as the mean ± SE (*n* = 3), and different letters indicate significant differences (*P* < 0.05).

It has been reported that H_2_S can directly affect the function of a protein through posttranslational modification^33^; therefore, to further evaluate how H_2_S regulates the activities of cell-wall-modifying enzymes, we next analyzed the effect of H_2_S on several key enzymes involved in the abscission process, namely, TAPG4 and Cel5, at the protein level. Both the TAPG4 and Cel5 proteins were exogenously expressed and purified (Fig. 4a), and the activity changes in these two proteins after treating explants with different concentrations of H_2_S (provided by NaHS), within the range of physiologically active concentrations, were monitored. We found that, when the concentration of the H_2_S donor (NaHS) reached 40 μM, the activity of Cel5 was only slightly reduced (Fig. 4b), while the activity of TAPG4 did not show any significant changes at the same concentrations of NaHS as those used in the petiole abscission assay (Fig. 4c). No significant correlation could be detected between the activity levels of either enzyme or the NaHS concentration.

### H_2_S affected the endogenous biosynthesis and/or signaling of ethylene and auxin

**Fig. 5:**
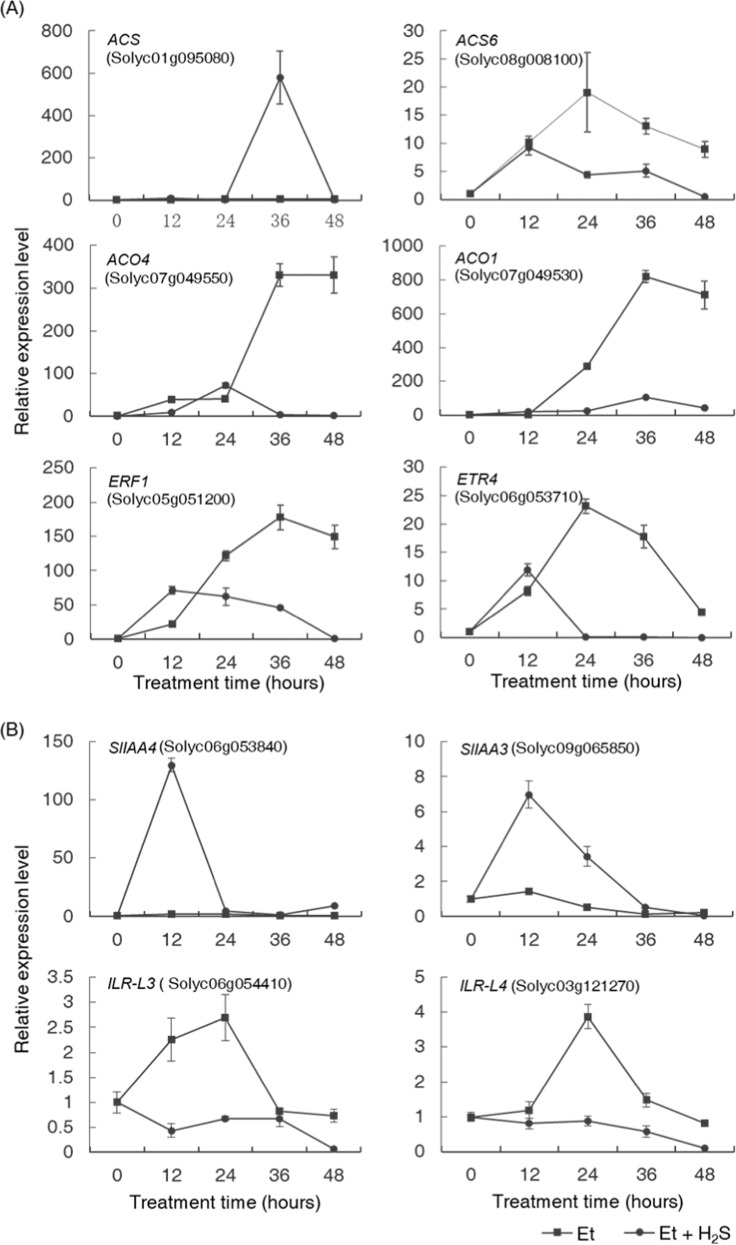
H_2_S affected the transcription of genes involved in the signaling of ethylene and auxin. Effect of H_2_S on changes in the transcription levels of **a** ethylene biosynthesis (*ACS*, *ACS6*, *ACO4*, and *ACO1*) or ethylene signaling-related (*ERF1* and *ETR4*) genes and **b** indicator genes of the bioactive-free auxin in the petiole abscission zone (AZ) during ethylene-induced abscission. mRNAs were extracted from AZ tissues under ethylene (squares) or ethylene–H_2_S (circles) treatments at the time points of 0, 12, 24, 36, and 48 h. Data are presented as the mean ± SE (*n* = 3). The *SAND* gene was used as an internal control.

Because H_2_S inhibited petiole abscission in the presence of ethylene, we wondered whether H_2_S interfered with the action of ethylene during this process. To test this hypothesis, we analyzed a number of key genes, namely, *ACS*, *ACS6*, *ACO1*, *ACO4*, *ERF1*, and *ETR4*, which had previously been shown to be involved in the biosynthesis or signaling of ethylene and to play essential roles during the process of abscission in tomato^31^. Real-time qPCR assays between the ethylene treatment group and the H_2_S–ethylene treatment group showed that H_2_S suppressed the upregulation of the transcription of the genes *ACS6*, *ACO1*, *ACO4*, *ERF1*, and *ETR4* in the late stages of the abscission process, especially after 24 h. However, the transcription of *ACS* was significantly increased by H_2_S, resulting in an expression peak at 36 h of treatment (Fig. 5a).

Auxin is also an essential hormone in the timing of the abscission process, and the level of auxin within the AZ tissues plays a crucial role in maintaining the insensitivity of AZ cells to ethylene and inhibits the occurrence of abscission. To assess the status of auxin in AZ tissues, the transcription levels of IAA/AUX family genes (*SlIAA4* and *SlIAA3*) and the genes from the IAA–amino acid conjugate hydrolase (ILR) family (*ILR-L3* and *ILR-L4*) were analyzed (Fig. 5b). The expression of these genes was an indicator of the auxin status in vivo. Our results showed that H_2_S significantly upregulated the expression of the *IAA*/*AUX* genes in the earlier stages of the abscission process, especially before 24 h after treatment. The transcription of the *ILR* family genes was downregulated in the H_2_S treatment group compared to the ethylene treatment group in the same time phase (Fig. 5b), suggesting an increase in the content of the bioactive-free auxin in the H_2_S-treated AZ tissues.

### H_2_S inhibited other cell separation processes in different plant species

**Fig. 6:**
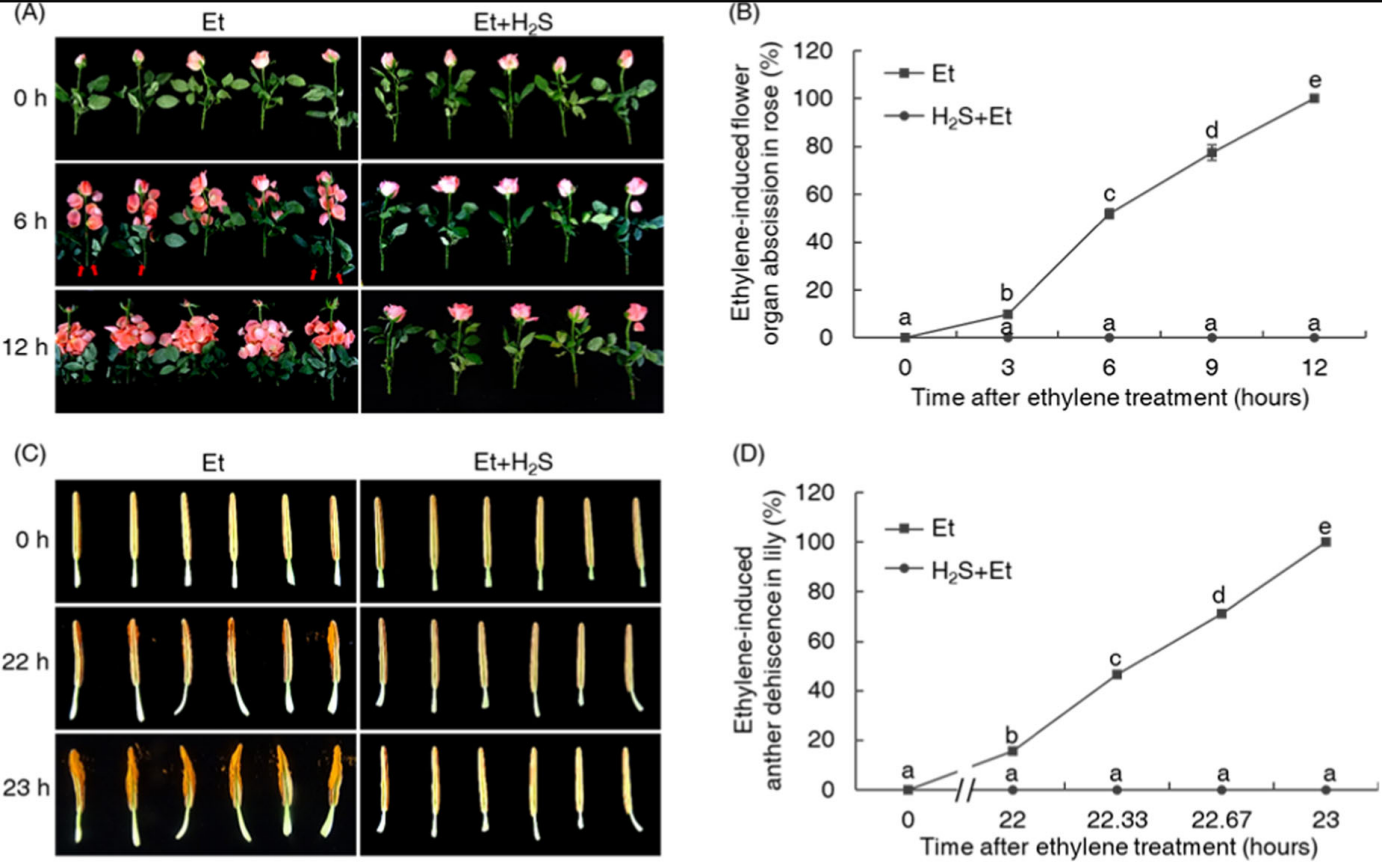
H_2_S affected the ethylene-induced cell separation processes in other plant species. H_2_S inhibited ethylene-induced floral organ abscission in rose (**a**, **b**) and anther dehiscence in lily (**c**, **d**). The rose flowers were treated with ethylene (provided by 40 μl of 1 M ethephon solution) or ethylene plus H_2_S (provided by 16 μl of 1 M NaHS solution) for 12 h, and the abscission of floral organs and leaves was photographed every 6 h and recorded every 3 h. Red arrows indicates the abscised leaves. The conditions used for the lily anthers were the same as those used for rose, and the dehiscence of lily anthers was monitored every hour after treatment. After the initiation of anther dehiscence in the ethylene-treated group, the dehiscence rates were recorded every 20 min.

To investigate whether H_2_S affected other abscission or cell separation processes in plants, we further analyzed the effects of H_2_S on floral organ abscission in rose and on anther dehiscence in lily. In the floral organ abscission assay, H_2_S (provided by NaHS solution) was applied, and ethylene (provided by ethephon solution) was also used to synchronize abscission. As shown in Fig. 6, half of the rose floral organs treated with only ethylene were abscised at 6 h after treatment (Fig. 6a, b), with all the petals abscised by the 12 h treatment time point. In addition, leaf abscission was also complete at 12 h of exposure to ethylene (Fig. S3). However, under H_2_S treatment, rose petal abscission did not start even at 12 h after the initiation of the H_2_S treatment (Fig. 6a, b). Moreover, H_2_S also inhibited the dehiscence of the anthers in lily. Anthers in the ethylene treatment group began to dehisce by 22 h of treatment, whereas in the H_2_S–ethylene treatment group, the anthers did not show any sign of dehiscence even at 23 h after the initiation of the treatment, by which time the anthers in the ethylene treatment group were already fully dehiscent (Fig. 6c, d).

## Discussion

### Hydrogen sulfide functioned as a novel regulator during the process of plant organ abscission

Abscission is a ubiquitous and dynamic process in the life cycle of plants. It helps plants defend themselves against adverse conditions by detaching damaged or infected organs, and it can also help plants disseminate their offspring through seed dispersal. In general, the very shape of plants is controlled by the processes of both growth and abscission^1^. In the past few decades, multiple phytohormones have been shown to be involved in the process of abscission, including ethylene^7–9^, auxin^11^, jasmonic acid^34^, and salicylic acid^35^. In modern agriculture production, many analogs or antagonisms of these hormones have also been applied in orchards to improve agricultural output or facilitate agricultural production activities^1^.

In the current study, we found that H_2_S, a newly discovered gasotransmitter^18^, also had a regulatory function during the abscission process at its physiologically active concentration (Fig. 1a). The exogenous application of H_2_S not only inhibited petiole abscission in tomato but also affected floral organ abscission in rose and anther dehiscence in lily (Fig. 6), indicating that the regulatory function of H_2_S with respect to the abscission process is widespread in the plant kingdom. In this work, we also found that the application of a H_2_S scavenger (HT) accelerated the process of abscission (Fig. 1a), indicating that, during the natural abscission process, endogenous H_2_S might also occupy an important position in the regulatory network.

Currently, several chemicals, which are inexpensive and easy to obtain^36^, have been shown to act as sources of H_2_S. As H_2_S at its physiological concentration also benefits human health^18^, it would be interesting to develop H_2_S as a new abscission regulator by manipulating exposure to H_2_S donor chemicals.

### H_2_S inhibited the activity of cell-wall-modifying enzymes by downregulating gene transcription

The increased activity of cell-wall-modifying enzymes, such as Cels and PGs, is an important indicator of the activation of abscission^37^. These enzymes work together to breakdown the adhesion between AZ cells and to promote cell separation. In the current study, we found that, compared with ethylene treatment alone, H_2_S treatment markedly suppressed the normal increase in the activities of both Cels and PGs (Fig. 2), and our research also revealed that H_2_S significantly inhibited the upregulation of genes encoding these enzymes during abscission (Fig. 3) but had little to no effects on these enzymes directly at the protein level via posttranslational modification (Fig. 4). These findings demonstrated that H_2_S probably functioned in achieving competence to respond to abscission signals or played a role in the transduction of abscission signals, as the transcriptional upregulation of genes encoding cell-wall-modifying enzymes indicates that the abscission signals had been passed far downstream and that the abscission process had been initiated^37–39^.

### H_2_S might maintain the insensitivity of AZ tissues to ethylene by modulating the content of bioactive-free auxin locally

As mentioned above, ethylene and auxin are important hormones regulating the process of abscission^9,11^. These two hormones have been suggested to work together in the timing of abscission^7^. To explore the mechanism underlying the inhibitory function of H_2_S during the process of petiole abscission in tomato, the expression changes in ethylene- and auxin-related genes were analyzed in this study. Our results showed that H_2_S interfered with ethylene signaling during abscission, as five out of the six genes examined (related to ethylene biosynthesis or signaling), which have been reported to be involved in the process of abscission, exhibited decreased transcription in response to H_2_S treatment compared to ethylene treatment^31,32^. Among the five regulated genes, three genes (*ACS6*, *ACO4*, and *ACO1*) were related to ethylene biosynthesis. Their expression peaked after 24 h, indicating that these genes were also involved in the phenomenon of ethylene autocatalysis, which occurred 24–48 h after ethylene treatment^40–42^. The regulatory effects of H_2_S on these genes also indicated that, in the petiole abscission process under ethylene treatment, H_2_S also affected the autocatalytic effect of ethylene. The transcriptional-level analyses of the other two ethylene signaling-related genes (*ERF1* and *ETR4*) showed that, under ethylene treatment, the expression of these two genes was upregulated significantly from earlier stages of abscission, and thus they might be involved in the signaling network that leads to the initiation of the abscission process. However, under H_2_S–ethylene treatment, the transcription of these two genes only began to decrease after 12 h, and after 24 h of treatment, the expression decline became significant in the H_2_S–ethylene treatment group compared to the ethylene treatment group (Fig. 5a). In the transcriptional analysis of the cell-wall-modifying-related genes under H_2_S treatment, we found that H_2_S inhibited the transcription of these genes from earlier stages of the abscission process (Fig. 3), indicating that the interference of H_2_S with ethylene signaling might not be the major function of H_2_S in abscission initiation. Regarding the inhibitory effect of H_2_S on these ethylene-related genes in later stages of abscission, future studies are needed to clarify whether H_2_S also has a role in the subsequent abscission processes.

The regulatory effects of H_2_S on auxin occurred in the earlier stages of abscission process. IAA/AUX proteins are repressors of the transduction of the auxin signal, but the transcription of some members of this family was always rapidly induced by the elevated free auxin level to ensure a strictly controlled transient response to changes in the auxin concentration through negative feedback^31,43^. Here two *IAA*/*AUX* family genes, which have been shown to respond to the changes in the auxin content rapidly^31^, were promoted dramatically in earlier stages of the abscission process after H_2_S treatment (Fig. 5b), suggesting an increase in the bioactive-free auxin in these H_2_S-treated AZ tissues. This deduction was further confirmed by the changing expression patterns of two *ILR* family genes. Enzymes encoded by this kind of genes promote the hydrolysis of IAA conjugates to release bioactive-free auxin^44^. The transcription of some genes from this family can be upregulated when the surrounding free auxin is deficient and the IAA conjugates are needed to be activated^31^. In this study, we found that the expression of two *ILR* genes was inhibited under H_2_S–ethylene treatment compared with ethylene treatment (Fig. 5b), indicating that the content of the free bioactive auxin in the H_2_S-treated AZ tissues was relatively high. As auxin has an important role in step 2 of abscission^7^, it is likely that H_2_S functioned in the second step of the abscission process by preventing the AZ cells from obtaining the competence to respond to abscission signals by modulating the content of the biologically active free auxin in these cells. Regarding the regulatory effect of H_2_S on ethylene-related genes in the abscission process after ethylene treatment, it is possible that H_2_S functioned by enhancing the content of bioactive auxin in AZ tissues, as it has been shown that IAA can inhibit the ethylene-regulated expression of ACO and ACS genes^45^. On the other hand, H_2_S might also have other ways to regulate the transcription of these genes because the expression of the ACS gene was enhanced by H_2_S in this analysis, and the inhibitory effect of auxin on this gene has previously been detected^27^. Thus our expression analysis results actually presented an integrated regulatory effect of H_2_S, and the exact regulatory mechanism underlying these phenomena still needs further elucidation.

### H_2_S functioned differentially in two ethylene-related physiological processes

The triple response is the typical phenotype after ethylene treatment, which consists of the exaggeration of the apical hook curvature and the thickening and shortening of hypocotyls and roots^46^. In this study, we found that, although H_2_S inhibited ethylene-induced petiole abscission in tomato (Fig. 1), this gasotransmitter showed no obvious effect on the triple response caused by the exogenous ethylene treatment (Fig. S2).

Regarding the functional discrepancy of H_2_S during these two processes, one possible explanation is that the regulatory mechanisms underlying these two processes are not identical, or in other words, they are mostly different. In the abscission process, it is well known that ethylene and auxin act antagonistically to regulate the timing of abscission^1^. In the triple response, evidence has shown that a partially synergistic relationship exists between these two hormones^46^, suggesting the complexity of the interaction of these two hormones in different physiological processes. On the other hand, it has recently been shown that the S-sulfhydration of the target proteins is an important mode of action for H_2_S^30^. As the components of both ethylene and auxin signaling pathways have expression preferences in different physiological processes^47^, it is possible that in the abscission process the upstream factors of the auxin signaling- and/or ethylene signaling-related genes could be modified and affected by H_2_S and thus affect the transcription of the downstream genes. However, in the triple response, no such factors exist, and no obvious effect could be detected after H_2_S treatment.

In addition to auxin and ethylene, H_2_S also interacts widely with other phytohormones, such as gibberellins, salicylic acid, and abscisic acid, in diverse processes during the development and stress responses of plants. It seems that H_2_S functions as a “referee” to harmonize the interaction between hormones and, as such, should be considered to be an integral molecule in the hormone signaling network of plants^48^. The determination of the detailed mechanism of the interactions between H_2_S and these phytohormones during the respective physiological processes will be valuable.

## Supplementary information

Primers used in this study

H2S inhibited the natural abscission of tomato petiole

H2S did not inhibit the triple response of ethylene in tomato seedlings

H2S inhibited the ethylene-induced leaf abscission in rose
