# Supplementary material for: Hydrogen sulfide inhibits ethylene-induced petiole abscission in tomato (Solanum lycopersicum L.)
Source: Hortic Res. 2020 Feb 1;7:14. doi: 10.1038/s41438-019-0237-0 (PMC6994592; doi:10.1038/s41438-019-0237-0)
Supplement: Supplementary file 1 — Primers used in this study [file 41438_2019_237_MOESM1_ESM.pdf]

TableS1 Primers used in this study

Assays	Destination products	Primer name	Primer sequence	Tm	product length
cloning, prokaryotic expression	pCold-CEL5	CEL5-BamH Ⅰ -F	GCTGGATCCATGGCTCATGCTCATGCTAA	75.5℃	1494 bp
		CEL5-Sal Ⅰ -R	GACGTCGACTTAATTTGGTGCGGATAAGA	70.7℃	
	pCold-TAPG4	TAPG4-BamH Ⅰ -F	GCTGGATCCATGAGTCCCTTAGCAATTTT	70.9℃	1164 bp
		TAPG4-Sal Ⅰ -R	GACGTCGACTTATAAGCATTTACTAAGTT	60.8℃	
qPCR	Cel5	Cel5-F	AATCGGAGCCTGCTACATAC	54.6℃	163 bp
		Cel5-R	CTACCACGGGACCATACTGA	55.5℃	
	TAPG4	TAPG4-F	CAATTAATATTCCAACTTCCCCT	56.9℃	188 bp
		TAPG4-R	TCGTAGAAGCACATGCAGAAC	56.3℃	
	TAPG2	TAPG2-F	CCTTCCACTATTAATGTACCAGC	55.8℃	198 bp
		TAPG2-R	GGTACCACTATAGATGGAAAGGG	56.9℃	
	Expansin 1	Expansin-F	ACAGCCAAGGATACGGAGTT	56.3℃	228 bp
		Expansin-R	CAAAGTGAGGGCGAGGAG	55.9℃	
	ACS	ACS-Q-F	GGATGATGGAACGGTTGATA	55.3℃	195 bp
		ACS-Q-R	AATGGTGAGGGAGGAATAGG	55.6℃	
	ACS6	ACS6-Q-F	AACCAAATAAGGGAGTTGAAGT	54.7℃	141 bp
		ACS6-Q-R	CTTGGACCATAGGAGAAGACA	54.2℃	
	ACO4	ACO4-Q-F	GATGGGACTCGGATGTCACTA	57.0℃	105 bp
		ACO4-Q-R	TTTCGGATAAACTTGCTTGCT	57.5℃	
	ACO1	ACO1-Q-F	CAATGAAGGCAATGGAAAGT	55.2℃	177 bp
		ACO1-Q-R	CATATGCAGCAAATCACAATC	54.1℃	
	ERF1	ERF1-Q-F	AAAAGATGTCAAGCCCACTAG	54.1℃	386 bp
		ERF1-Q-R	AAATGTTCCTAACCAAACCCTA	55.6℃	
	ETR4	ETR4-Q-F	ATGTAACTGTGATGATGAGGGAT	55.4℃	330 bp
		ETR4-Q-R	AGCAGCATAGGAAAGAGGGTA	56.3℃	
	SAND	SAND-Q-F	TTGCTTGGAGGAACAGACG	56.6℃	164 bp
		SAND-Q-R	GCAAACAGAACCCCTGAATC	56.9℃	
