# Supplementary figures and images for: Hydrogen sulfide inhibits ethylene-induced petiole abscission in tomato (Solanum lycopersicum L.)
Source: Hortic Res. 2020 Feb 1;7:14. doi: 10.1038/s41438-019-0237-0 (PMC6994592; doi:10.1038/s41438-019-0237-0)

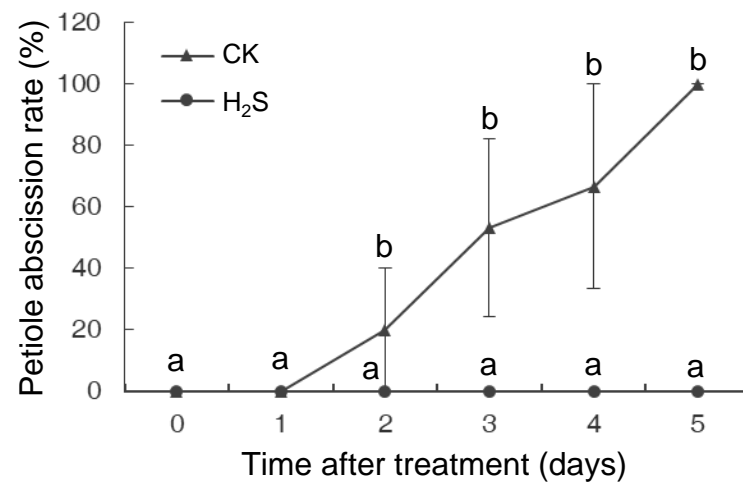

Figure S1

Supplement: Supplementary file 2 — H2S inhibited the natural abscission of tomato petiole [file 41438_2019_237_MOESM2_ESM.pdf]

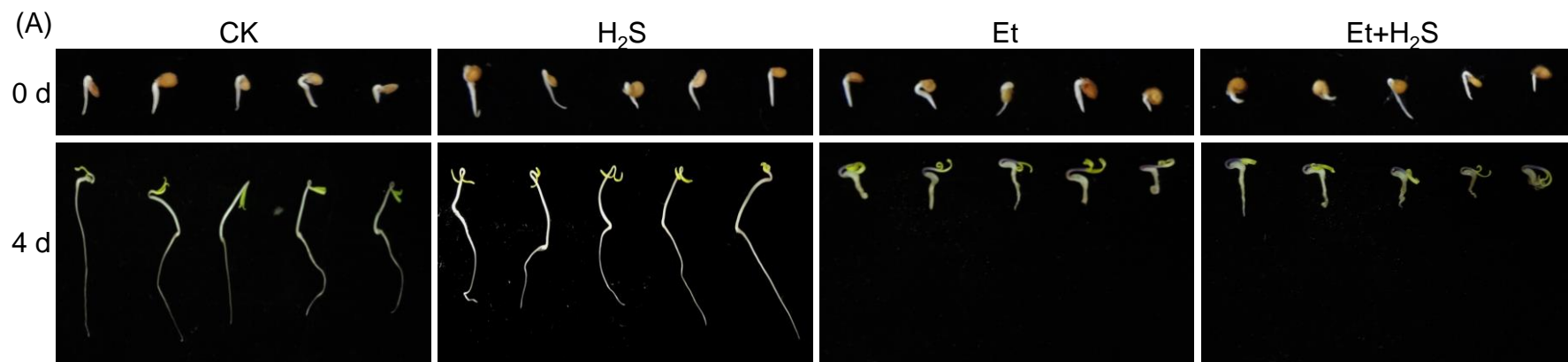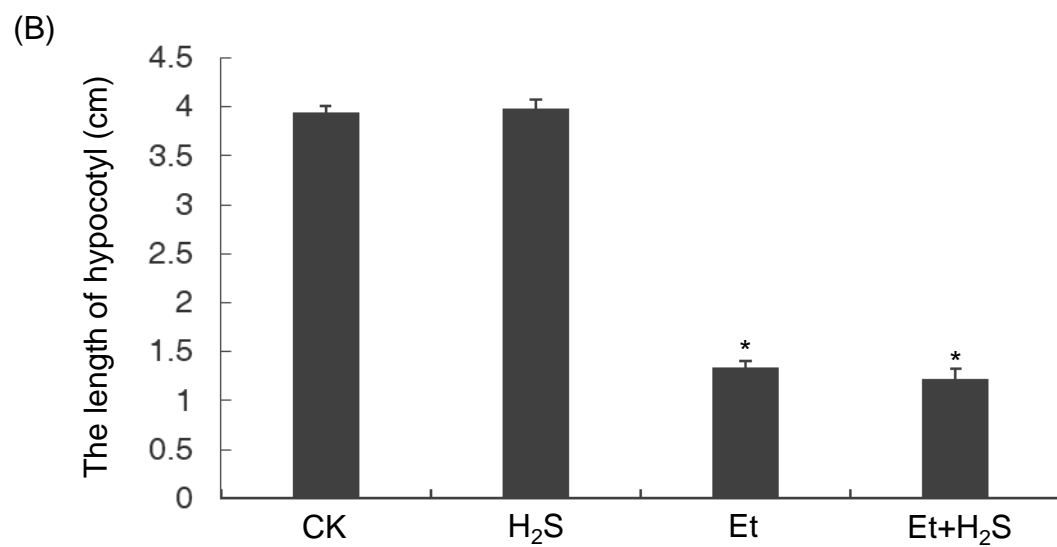

Figure S2

Supplement: Supplementary file 3 — H2S did not inhibit the triple response of ethylene in tomato seedlings [file 41438_2019_237_MOESM3_ESM.pdf]

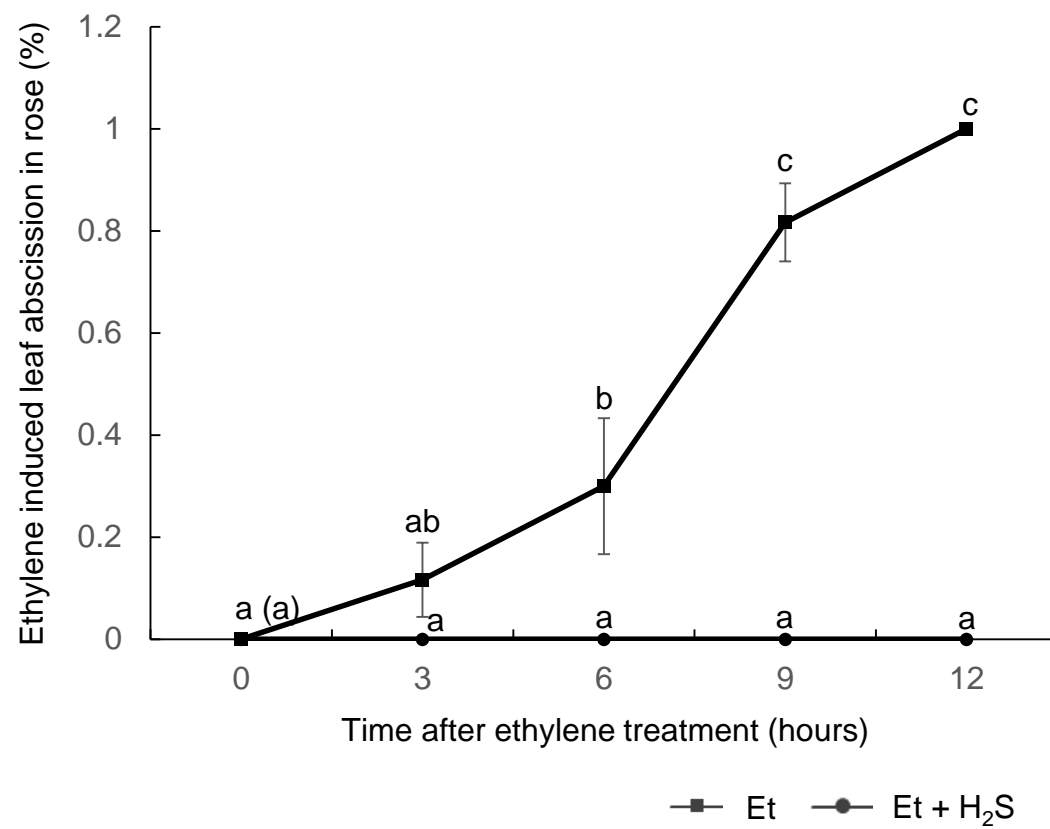

Supplement: Supplementary file 4 — H2S inhibited the ethylene-induced leaf abscission in rose [file 41438_2019_237_MOESM4_ESM.pdf]
